# Special Issue “Plant Responses to Abiotic and Biotic Stresses”

**DOI:** 10.3390/ijms26199651

**Published:** 2025-10-03

**Authors:** Longxing Tao, Tingting Chen

**Affiliations:** State Key Laboratory of Rice Biology and Breeding, China National Rice Research Institute, 359 Tiyuchang Road, Hangzhou 310006, China; taolongxing@caas.cn

During their growth and development, plants often encounter a variety of biotic and abiotic stresses, such as drought, high salinity, extreme temperatures, heavy metal pollution, and pests and diseases. These stresses disrupt the normal physiological metabolism of plants, leading to inhibited growth, reduced yield, and even death [1,2]. In agricultural production, these stress factors directly affect food security. For instance, globally, crop yield reduction caused by drought reaches 10–30% every year, while losses resulting from pests and diseases amount to 20–40%. In recent years, agricultural stress events have occurred with increasing frequency, posing severe multidimensional hazards to agricultural production [3]. Global warming has led to an increase in extreme weather events. Data from the World Meteorological Organization shows that the period from 2015 to 2024 was the warmest decade on record, and 2024 was the hottest year ever recorded globally, with the average global surface temperature being approximately 1.5 °C higher than that before the industrial revolution [4]. Under such environments, meteorological fluctuations have intensified significantly; extreme weather events have occurred more frequently, heatwaves have become more common, and phenomena such as alternating freezing and drought periods, as well as sudden shifts between droughts and floods, have happened repeatedly [5]. Moreover, the range of pests and diseases has expanded. Climate warming has reduced the mortality rate of overwintering pests and diseases, significantly increasing the base number of surviving pests and diseases after winter. This has led to an increase in the number of generations of pests and diseases, an extension of their damage period, and the expansion of their infestation range to the north. In the past, the cotton bollworm rarely appeared in the cotton-producing areas of North China, but after climate warming, it broke out on a large scale in this region. There have also been extreme shifts between droughts and floods. Global warming has intensified the transition between droughts and floods. Taking the Poyang Lake Basin as an example, the water area reached a historical maximum in 2020. However, within just two years, by 2022, the basin experienced an extreme drought event that resulted in a severe contraction of the lake area, significantly impacting agricultural activities in the surrounding regions.

The hazards of stress factors to agricultural production are manifested in the following aspects. Firstly, there is a significant reduction in crop yield [6]. From 2023 to 2024, Ghana experienced droughts and high temperatures, which created harsh growth conditions for cocoa trees and exacerbated pests and diseases. As a result, cocoa production plummeted by 40% from the expected 820,000 tons to less than 500,000 tons. In May 2024, due to heavy rain and flooding in Rio Grande do Sul, Brazil, the soybean yield of this season dropped from the predicted 22.24 million tons to 19.53 million tons, with a loss of approximately 10% to 15%. In 2023, Henan Province was hit by “harvest-season rain” (prolonged rain during the wheat harvest period), which caused wheat lodging, germination, and mildew, leading to a nearly 6.9% year-on-year decline in summer grain yield. Secondly, the quality of agricultural products is impaired [7]. In September 2024, the grape-growing area at the eastern foot of Helan Mountain in Ningxia experienced rare, continuous rainy weather for half a month. The sugar content of grapes dropped from the normal 230–240 g per liter to below 220 g per liter. This not only reduced the alcohol content of wine and decreased flavor substances but also caused grape cracking and the spread of pests and diseases due to the rain. In Thailand, long-term high temperatures of around 40 degrees Celsius have restricted the growth of durians, resulting in reduced durian yield, increased planting costs, and a shortened harvesting period. Thirdly, the farmland ecosystem is damaged [8]. Extreme weather conditions can cause soil degradation and soil erosion, exerting a negative impact on the farmland ecological environment. For example, long-term drought reduces the activity of soil microorganisms, destroys the soil aggregate structure, and leads to a decline in soil fertility. During floods, the scouring effect of floodwater on the topsoil is significant, thinning the soil layer and causing a large loss of nutrients. Furthermore, the agricultural product supply chain is disrupted. Extreme weather can also damage agricultural infrastructure, disrupt transportation and distribution, break the agricultural product supply chain, trigger market supply shortages, drive up prices, and exacerbate inflation. During the 2025 floods in South China, logistics in major banana-producing areas were interrupted, leading to a 45% weekly increase in the market price of bananas. To address the challenge of such stresses, it is crucial to understand how plants effectively cope with high-intensity extreme weather conditions, and to elucidate the underlying molecular and physiological mechanisms governing their responses to abiotic stresses [9,10]. Thus, we organized a Special Issue titled “Plant Responses to Abiotic and Biotic Stresses”. This issue comprises fifteen contributions that supply novel insights into the mechanisms underlying plants’ responses to abiotic and biotic stresses. It is precisely this knowledge that can facilitate the development of technologies to assist plants in withstanding abiotic and biotic stresses, thereby alleviating the impacts of external stresses and ensuring crop security [11,12].

Niedziela et al. (Contribution 1) studied Aluminum (Al) stress and its relation to epigenetic mechanisms in triticale, whose stress tolerance is linked to *ALMT* (7R) and QTLs (3R, 5R, 6R) with 36% heritability. The results showed that different methods for quantifying Al-induced DNA methylation changes led to detailed differences, and DN-CG, DM-CG, and DN-CHG markers were key to explaining Al tolerance. Additionally, they found that epigenetic markers had different densities but comparable distribution patterns in root tip and leaf tissues (supporting Al stress transmission via somatic memory), and that methylation changes were genome-wide, unrelated to Al stress-specific genes. Thus, the study indicated that Al stress may spread through somatic memory, with its induced methylation changes occurring across the genome rather than targeting specific Al-related genes.

Research by Ming et al. (Contribution 2) provides the first genomic identification of SPL transcription factors in *Ginkgo biloba*. Thirteen *GbSPL* genes were identified and phylogenetically grouped. Dispersed duplication contributed to family expansion. Promoter analysis indicated roles in flavonoid biosynthesis and stress adaptation via light, hormone, and stress-responsive elements. Expression analyses showed tissue-specific patterns, differential expression in flavonoid-rich leaves, and regulation under water stress. Nuclear localization of GbSPL2/11 was confirmed. This study establishes a functional and evolutionary foundation for GbSPLs in secondary metabolism and drought response.

Gaonosi et al. (Contribution 3) found that *Aphis craccivora* reduces cowpea yield. This study evaluated host-plant resistance. Tswana and B261-B were resistant, with low aphid growth and damage. Tswana likely suppressed aphid reproduction. Several genotypes were susceptible, showing high aphid counts, sooty mold, and plant damage. Resistance markers SNP1_0912 and CP 171F/172R validated resistance in Tswana and ER7, but failed in some controls, possibly due to QTL–environment interactions or aphid biotypes. Findings support breeding resistant cowpea lines; Tswana will be sequenced to develop new markers.

Ginori et al. (Contribution 4) evaluated heat and light stress tolerance in four wax begonia genotypes. Tolerant types (FB08-059, OPGC 5104) exhibited thicker cuticles, acute leaf folding, and elevated anthocyanin, aiding photoprotection. FB08-059 showed a 25.83% cuticle increase and 3× anthocyanin rise, limiting ROS and sustaining photosynthesis. Susceptible genotypes (Cocktail Vodka, Sprint White) had higher ion leakage, chlorophyll loss, and impaired gas exchange. Findings of the research highlight cuticle thickness, anthocyanin accumulation, and leaf folding as key indicators of heat and light stress resilience, and could support breeding programs for stress-resilient cultivars.

Yuan et al. (Contribution 5) identified 72 *CaBTB* genes in pepper (*Capsicum annuum* L.), revealing phylogenetic collinearity with related species. Expression and RT-qPCR analyses showed that multiple *CaBTB* genes responded significantly to various treatments. *CaBTB25* was highly expressed in leaves, contained light-responsive promoters, and was localized in the nucleus without transcriptional activation. These results provide a foundation for understanding *CaBTB* gene functions in pepper development and environmental interactions.

In *Eucalyptus grandis*, twelve phosphate starvation responses (EgPHRs) were identified by Xu et al. (Contribution 6) and phylogenetically classified into three groups. EgPHRs form a species-specific interaction network, with EgPHR6 binding multi-species SPX proteins, while woody plant PHRs uniquely interact with TRARAC-kinesin ATPase. Promoter analysis revealed 59 transcription factors regulating EgPHR expression linked to development, stress, and hormones. Transcriptomic and RT-qPCR analyses showed that all EgPHRs respond to phosphate deficiency, with EgPHR2/6 strongly induced; they also react to salt, cold, jasmonic acid, and boron deficiency. Notably, nitrogen starvation suppressed most EgPHRs, indicating nutrient signaling crosstalk. These results underscore EgPHRs’ multifaceted role in abiotic stress adaptation in woody plants.

Chlorine (Cl), an essential plant nutrient, is underutilized in agriculture due to toxicity concerns at high levels. The study of Su et al. (Contribution 7) identifies 3 mM as the optimal Cl^−^ concentration in nutrient solutions, significantly enhancing tomato plant biomass, yield, and chlorophyll content. Applying 3 mM Cl^−^ (as CaCl_2_ and KCl) boosted photosystem I and II activities, increasing the net photosynthetic rate by over 25%. In fruits, Cl^−^ elevated soluble sugars by 12.3 to 16.5% by promoting glucose, fructose, and sucrose accumulation. This resulted from enhanced invertase activity, sucrose synthase, and sucrose phosphate synthase during ripening. The findings reveal the critical role of Cl^−^ in improving fruit quality and photosynthesis, providing valuable insights for optimized fertilization in protected horticulture.

Cyanobacteria from extreme environments are biotechnologically valuable due to their adaptability and natural products. Bilova et al. (Contribution 8) compared the primary metabolomes of twelve such strains using GC-MS/LC-MS. Results showed distinct metabolite profiles across all strains, with greater differences among those from highly contrasting habitats. Terrestrial and freshwater extremely tolerant strains showed fewer uniquely accumulated metabolites than extremophiles from saline–alkaline environments, which displayed high metabolic diversity. This suggests divergent survival mechanisms in high-salinity/alkalinity habitats. Strain-specific metabolites are linked to both persistence during storage and original environmental adaptations.

Drought stress severely impacts the growth, quality, and yield of *Codonopsis pilosula* seedlings. Wang et al. (Contribution 9) evaluated nine cultivars under well-watered (BF) and drought-stressed (SF) conditions using a growth–physiology–yield composite and drought tolerance indices. Water treatment significantly affected emergence rate, while both water treatment and cultivar influenced root length, proline, ascorbate peroxidase activity, and chlorophyll content. Cultivar G1 outperformed others across multiple metrics. Drought stress reduced total root length, single root fresh weight, and yield by 18.33%, 28.4%, and 33.9%, respectively. Cultivars exhibited distinct physiological responses to drought, with varying tolerance levels identified. The findings support breeding efforts for drought-resistant cultivars of *C. pilosula*.

Teng et al. (Contribution 10) examined temperature effects on polyamine metabolism in the diatom *Skeletonema dohrnii*. Under three temperature regimes, physiological parameters, polyamine composition, and polyamine oxidase (PAO) gene expression were analyzed. Low temperatures reduced growth rate, increased biogenic silica, raised putrescine and spermine content, decreased spermidine, and down-regulated PAO expression. High temperatures increased growth rate and spermidine, and altered putrescine and spermine concentrations. The findings indicate that temperature shifts influence algal growth and silica content, induce specific polyamine profile changes, and implicate PAO gene involvement in temperature response regulation.

Spychala et al. (Contribution 11) investigated the molecular basis of adult plant resistance (APR), including miRNA expression and their target genes, in wheat hybrids derived from resistant cultivar Glenlea and Polish varieties. Under controlled inoculation with *Puccinia triticina* (Pt), RT-qPCR analysis revealed time-specific differential expression of APR genes and miRNAs, with peak expression at 6, 24, and 48 h post-inoculation. Notably, *Lr46-Glu2* was upregulated, indicating its role in the immune response. Gene ontology analysis identified hundreds of potential targets for tae-miR5384-3p and tae-miR9653b, advancing understanding of APR-based defense mechanisms. The results reveal that wheat APR genes (e.g., Lr46) and miRNAs collaboratively regulate resistance to leaf rust through temporal expression.

Xiong et al. (Contribution 12) found that nano-silicon foliar application alleviates salt stress in rice seedlings (variety 9311) by improving root architecture, enhancing photosynthesis, boosting antioxidant enzyme activities, increasing hormone contents of indole-3-acetic acid, cytokinin, gibberellin, jasmonic acid, salicylic acid, reducing abscisic acid and ROS, promoting Si^4+^, K^+^, and Ca^2+^ uptake, and decreasing Na^+^ and Cl^−^ accumulation, ultimately supporting better growth under NaCl stress.

Li et al. (Contribution 13) identified 122 subtilase (*SBT*) genes in cultivated peanut (*Arachis hypogaea* L.), classifying them into six phylogenetic subgroups. Structural and collinearity analyses revealed evolutionary conservation, with numerous homologs in *Glycine max*. Cis-element, GO, and KEGG analyses indicated roles in hormone response and abiotic stress. Tissue-specific expression profiling highlighted genes like *AhSBT2* and *AhSBT102* in seed development, while *AhSBT39* and AhSBT115 were upregulated under drought, cold, hormone treatments, and *Ralstonia solanacearum* infection in resistant varieties. The results provide foundational insights into *AhSBT* functions for improving stress tolerance and agronomic traits in peanut.

Tobiasz-Salach et al. (Contribution 14) investigated the role of foliar silicon (Si) application in alleviating salt stress (200 mM NaCl) in wheat. Si at 0.1% and 0.2% concentrations significantly improved physiological parameters, including chlorophyll content (CCI), gas exchange (*P*_N_, *C*_i_, *E*, *g*s), and chlorophyll fluorescence (PI, RC/ABS, F_V_/F_m_, Fv/F_0_), compared to the lower dose (0.05%). The mitigating effect was sustained over time. Additionally, MSAP analysis revealed that Si application induced dose-dependent changes in DNA methylation patterns, suggesting epigenetic regulation as a key mechanism in enhancing salt adaptation. These findings demonstrate that foliar Si can effectively mitigate salinity impact through physiological and epigenetic modifications.

Wang et al. (Contribution 15) explored mechanisms behind the balance between high yield and quality by using two varieties of ZZY1 and ZZY8. ZZY1 showed higher yield due to greater seed-setting and grain weight, while ZZY8 exhibited superior quality with lower chalkiness and higher head rice rate. Although ZZY1 had lower total dry matter, it allocated more to panicles and nonstructural carbohydrates, with higher grain-filling rates, ATP/ATPase levels, and key enzyme activities early in filling. Transcriptome analysis highlighted carbohydrate and energy metabolism as central to trade-offs, indicating that limited energy in ZZY1 hindered simultaneous high yield and quality development.

Plant responses to abiotic and biotic stresses involve intricate physiological adjustments, molecular regulation, and epigenetic modifications, as revealed by fifteen studies focusing on crops like rice, wheat, peanut, etc. The abiotic stresses involved in our thematic study include water/drought stress, salt stress, aluminum and chloride ion stress, heat and light stress. In addition, three studies were conducted from the aspect of genome-wide identification and analysis, and one study was carried out from the aspect of primary metabolome analysis. Wang et al. (Contribution 15) linked rice yield-quality discordance to energy metabolism genes. Through these abiotic stress research, epigenetic regulation is deepened. The study on triticale under aluminum stress compares three DNA methylation quantification models, identifying DN-CG, DM-CG, and DN-CHG as core epigenetic markers. These markers show similar distribution in roots and leaves, supporting somatic memory-mediated stress signal transmission, beyond prior focus on genetic variation. Transcription factor functional expansion is notable and morpho-physiological mechanisms are refined. In *Ginkgo biloba*, 13 *GbSPL* genes were found to expand via dispersed duplication, with only 2 miR156-targeted, differing from angiosperms. GbSPL2/11 localize to the nucleus, regulating flavonoid biosynthesis and water stress responses. Stress-tolerant *Begonia semperflorens* (FB08-059) uses 25.83% thicker cuticles, leaf folding, and tripled anthocyanins to maintain photosynthesis, providing new resistance evaluation indices. Research on biotic stress, as well as the study on the miRNA–gene regulatory network of wheat resistance to leaf rust, cover the precise excavation and marker validation of pest resistance resources. In summary, these studies break through the limitation of the “single stress–single gene–single mechanism” in traditional stress research. From multiple dimensions, such as transcriptional regulation and morphological–physiological integration, they construct stress response networks with both species-specificity and universality, providing new genetic resources and theoretical basis for breeding stress-resistant crops.
